# Health belief model for empowering parental toothbrushing and sugar intake control in reducing early childhood caries among young children—study protocol for a cluster randomized controlled trial

**DOI:** 10.1186/s13063-022-06208-w

**Published:** 2022-04-12

**Authors:** Ketian Wang, Gillian Hiu Man Lee, Pei Liu, Xiaoli Gao, Samuel Yeung Shan Wong, May Chun Mei Wong

**Affiliations:** 1 Division of Applied Oral Sciences and Community Dental Care, Faculty of Dentistry, The University of Hong Kong, Pok Fu Lam, Hong Kong; 2 Division of Paediatric Dentistry & Orthodontics, Faculty of Dentistry, The University of Hong Kong, Pok Fu Lam, Hong Kong; 3 Faculty of Dentistry, National University of Singapore and Saw Swee Hock School of Public Health, National University of Singapore, Queenstown, Singapore; 4 JC School of Public Health and Primary Care, Faculty of Medicine, The Chinese University of Hong Kong, Pok Fu Lam, Hong Kong

**Keywords:** Oral health education, HBM, ECC, RCT, mHealth

## Abstract

**Background:**

It has been recognized that oral health education for parents is critical for preventing early childhood caries (ECC). Few parents practiced caries prevention procedures for their children in daily life, though. A novel intervention scheme using mobile messages will be developed in this study under the framework of the health belief model (HBM). The objective of the present randomized clinical trial (RCT) is to evaluate the effectiveness of the new scheme in promoting oral health of young children by reducing dental caries.

**Methods:**

This RCT will involve 26–36 child care centers or kindergartens with nursery classes (clusters) located in Hong Kong. A total of 518–628 child-parent dyads (child age 18–30 months) will be recruited and randomly allocated at the cluster level into the test or control group with a 1:1 ratio. For parents in the test group, the intervention will consist of a set of HBM-based text messages sent regularly in 48 weeks. A standard text message will be sent to the parents in the control group in the first week. The primary outcome will be dental caries measured by dmft/dmfs of the children after 2 years (around 4 years of age). The secondary outcomes will be toothbtushing and sugar intake.

**Discussion:**

HBM-based intervention via a low-cost text messaging vehicle may serve as a viable way to empower parents to establish proper oral health behaviors for their children and safeguard the oral health of children in Hong Kong.

**Trial registration:**

ClinicalTrials.govNCT04665219. Registered on 11 December 2020.

**Supplementary Information:**

The online version contains supplementary material available at 10.1186/s13063-022-06208-w.

## Administrative information

Note: The numbers in curly brackets in this protocol refer to SPIRIT checklist item numbers. The order of the items has been modified to group similar items (see http://www.equator-network.org/reporting-guidelines/spirit-2013-statement-defining-standard-protocol-items-for-clinical-trials/).

**Table:** 

Title {1}	Health belief model for empowering parental toothbrushing and sugar intake control in reducing early childhood caries among young children—a cluster randomized controlled trial
Trial registration {2a and 2b}.	The trial is registered on ClinicalTrials.gov (NCT04665219) All items from the WHO Trial Registration Data Set can be found in the present protocol.
Protocol version {3}	Version no.3, dated 2021.03.30
Funding {4}	Health and Medical Research Fund (Project no.: 17181971), Food and Health Bureau (FHB), Government of Hong Kong SAR, China
Author details {5a}	MCMW conceived of the study. MCMW, HMGL, PL, XG, and SYSW initiated the study design, and KW helped with the implementation. MCMW, HMGL, PL, XG, and SYSW are grant holders. MCMW provided statistical expertise in the clinical trial design. All authors contributed to the refinement of the study protocol and approved the final manuscript.
Name and contact information for the trial sponsor {5b}	N/A
Role of sponsor {5c}	N/A

## Introduction

### Background and rationale {6a}

Dental caries is one of the most common chronic diseases during childhood. According to the Global Burden of Disease Study in 2017, more than 530 million children globally have dental caries of the primary teeth [1]. The latest oral health survey in Hong Kong found that 51% of children aged five had dental caries [2], and another study reported that 31% of children aged three had dental caries already [3]. Early childhood caries (ECC) is denoted as any form of caries occurring in the primary dentition of children aged 71 months or younger [4, 5]. ECC not only affects children’s oral health function, but also puts these children at greater risk of developing caries in the permanent dentition and results in lifelong impacts [4, 5]. Therefore, preventing decay experience in primary teeth would enhance children’s oral health-related quality of life and result in significant savings on dental service costs in the future.

Parents play an important role in shaping their children’s oral hygiene practices and eating habits from a very young age [6], which were strong risk factors for dental caries [7]. It has been recognized that oral health education for parents is critical for the prevention of ECC [8, 9]. Unfortunately, although large quantities of oral health education campaigns have been launched, few parents are conducting ideal caries prevention practices for their children in daily life [10–12].

A limited amount of information, engagement, and support can be conveyed by conventional health education approaches such as conversations or brochures. Mobile technologies are therefore popularized for their informativeness and interactivity, among which sending text messages has been proven effective in health-related behavior intervention [13]. Some studies indicated that sending text messages (SMS) was an effective method in oral health education among parents of preschool children [14, 15]. An ongoing trial is now evaluating the effectiveness of SMS as an adjuvant method for preventing ECC [16]. However, the age of children engaged in these studies varies, some of which could have missed the best time for ECC prevention [17]. Therefore, the present study targets younger children and aims to empower parents to establish proper oral health habits in their children.

Health belief model (HBM) is a social psychological model for health behavior change [18]. HBM-based intervention has been introduced to the field of dentistry to some degree. A recent systematic review indicated that HBM has a significant effect on improving the oral health of school children and adolescents [19]. No reported trial is available on how HBM-based intervention can be used among parents for promoting caries preventive behaviors in their infants. In this context, the effectiveness of HBM-based behavior intervention by text message is to be tested.

### Objectives {7}

The objective of this study is to investigate the effectiveness of the HBM-based behavioral intervention using SMS to promote parental oral health care behaviors (toothbrushing and sugar intake control) and reduce ECC compared to conventional oral health education.

Hypotheses to be tested are as follows:

i. The proposed HBM-based behavioral intervention via SMS will reduce ECC at around age 4 compared to conventional oral health education.
ii. The proposed HBM-based behavioral intervention via SMS will promote parental oral health care behaviors (toothbrushing and sugar intake control) for their young children more than conventional oral health education.

### Trial design {8}

**Fig. 1:**
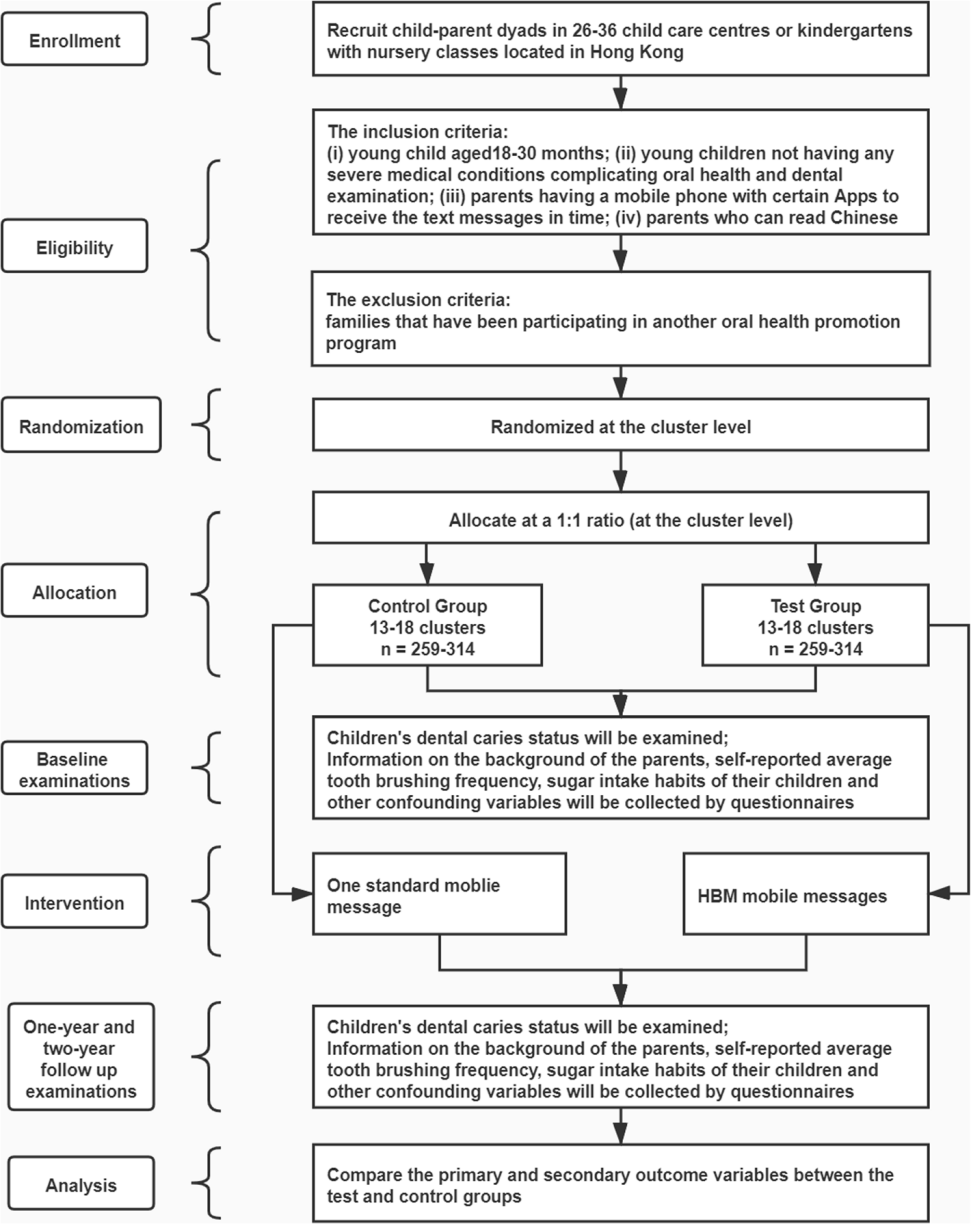
Study flowchart

**Fig. 2:**
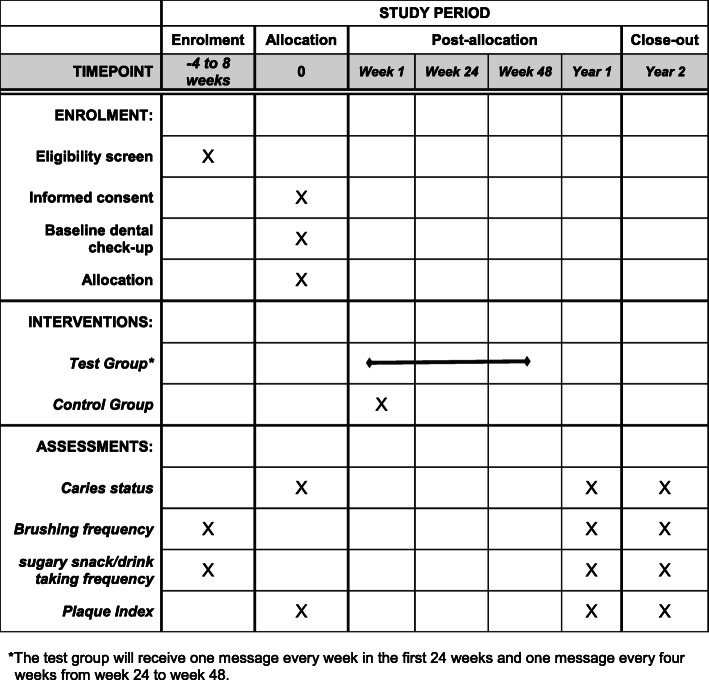
Participant timeline. Schedule of enrollment, interventions, and assessments. *The test group will receive one message every week in the first 24 weeks and one message every 4 weeks from week 24 to week 48

This study will be a two-arm parallel design cluster randomized controlled trial. The study design and the participant timeline are illustrated in Figs. 1 and 2. This RCT will be conducted according to the ICH-GCP and CONSORT Checklist [20, 21]. The ethical approval is obtained from the Institutional Review Board of the University of Hong Kong/Hospital Authority Hong Kong West Cluster (IRB: UW 20–029).

Hypotheses to be tested are as follows:

i. The proposed HBM-based behavioral intervention via SMS will reduce ECC at around age 4 compared to conventional oral health education.
ii. The proposed HBM-based behavioral intervention via SMS will promote parental oral health care behaviors (toothbrushing and sugar intake control) for their young children more than conventional oral health education.

## Methods: participants, interventions, and outcomes

### Study setting {9}

Child care centers and kindergartens with nursery classes, which formed the clusters of the trial, will be approached and invited to participate in the clinical trial. Each cluster will be considered as a unit for randomization and intervention.

### Eligibility criteria {10}

The target population will be young children and their parents (or primary caregivers). Child-parent dyads will be recruited.

The inclusion criteria will be as follows: (i) child aged 18–30 months, (ii) child not having any severe medical conditions complicating the oral health and dental examination, (iii) a parent having a mobile phone with certain apps to receive the text messages in time (e.g., WhatsApp or WeChat), and (iv) a parent who can read Chinese.

Families that have been participating in other oral health promotion programs will be excluded.

### Who will take informed consent? {26a}

The staff in those participating child care centers or kindergartens with nursery classes will help invite parents of the children to participate in the study and distribute information sheets and consent forms to the parents. All child-parent dyads who meet the inclusion criteria and are not in any other oral health programs will be invited to participate. The parents will be asked to bring the consent form to the kindergarten or the hospital to get the baseline check-up if they are willing to participate. The parent, the witness, and the researcher will sign the consent form before the baseline dental check-up.

### Additional consent provisions for collection and use of participant data and biological specimens {26b}

There are no additional plans to collect or use participant data.

### Interventions

#### Explanation for the choice of comparators {6b}

The study adopts an active control group to verify the effectiveness of the HBM framework in health promotion messages.

Parents in the control group will receive an oral health education pamphlet (in Chinese) produced by the Oral Health Education Division in the Department of Health, the Government of Hong Kong SAR [22], which is the standard dental care for young children in Hong Kong now. In order to maintain a double-blind design, the e-pamphlet will be distributed in the form of a mobile message. The participants will be told that they will receive some oral health information via the mobile message, without the content and the number of the message, so that they have no idea about the grouping. The selection of comparator is therefore justified.

#### Intervention description {11a}

The intervention in the test group will consist of a set of text messages developed based on the HBM to be sent to the parents regularly in 48 weeks.

According to the concept of HBM, parents are likely to adhere to recommended oral health care for children under a specific five sets of conditions [23]. First, parents must have some minimal level of knowledge about early childhood caries and motivation towards keeping their children caries-free. Second, parents must perceive the high caries risk of young children without good oral health care, and they must also be convinced that caries is a serious oral health problem for children, which could affect their general health. Third, parents must also be convinced that regular toothbrushing and control of sugar intake for their children are effective in preventing caries. Fourth, internal or external stimulus, referred to as “cue to action,” that triggers parental oral health care behavior in their children is present. Finally, parents’ self-efficacy to follow oral health care guidelines should be established and maintained during childhood.

The text messages to be sent will be designed and targeted on the six domains guided by HBM. We have gathered the questions, inquiries, comments, and feedback from the parents in our two recently completed clinical trials for the development of the messages.

While the set of standardized messages can be sent to tackle several HBM domains (susceptibility, severity, benefit), “cues to action” and “barriers” are likely to vary across different parents and life scenarios and might emerge anytime in the behavioral change process. Taking the advantage of interactivity (two-way communication) of text messaging, parents will be encouraged to share their concerns/experiences/thoughts via texting us back, which will be responded/discussed/solved promptly. By doing this, continuous support can be provided to facilitate the enhancement of parental self-efficacy (another HBM domain), through which positive actions are likely to take place.

During the first 24 weeks, all parents will receive a text message (and feedback if deemed appropriate) each week. In the next 24 weeks, the parents will receive a text message every 4 weeks; altogether, 30 messages will be sent to the parents. All messages will be sent to the parents individually through a free mobile app (e.g., WhatsApp or WeChat) by a research assistant. The RA (dentally trained) will seek consultation from the two dentists in our research team whenever necessary before responding to the parents.

#### Criteria for discontinuing or modifying allocated interventions {11b}

Since the study intervention is a mobile message, which will not directly affect the participant’s health status, the intervention will only be stopped when the participant withdraws from the study.

#### Strategies to improve adherence to interventions {11c}

Participation will be incentivized by offering participants a set of children’s oral health care products upon the completion of the assessment each time. Besides, a cash allowance of HK$100 will be offered to the parents to compensate for the time spent, any inconvenience caused, and cover transportation costs or other expenses that may incur for each visit to the Prince Philip Dental Hospital.

#### Relevant concomitant care permitted or prohibited during the trial {11d}

During the research period, all the participants will not be prohibited from any dental visits for check-ups or treatment. Information on their dental visits will be collected at each follow-up assessment.

#### Provisions for post-trial care {30}

If a child was found to have dental caries or other oral diseases during the dental check-up(s), the parent will be informed and be suggested to bring the child to a dentist. No additional intervention will be delivered to the control group after the trial.

### Outcomes {12}

The outcomes of this study will be composed of one primary outcome and three secondary outcomes, which have been illustrated in Table 1.

**Table 1:** The primary and secondary outcomes

Outcome		Elements				
		Domain	Specific measurement	Specific metric	Method of aggregation	Time-points
Primary	Dental caries status	Caries	dmft/dmfs	Change from baseline	Mean	1 year; 2 years
Secondary	Brushing frequency	Oral health behavior	Twice daily or more Once daily 4 times or more weekly 2–3 times weekly Once or less weekly	Change from baseline	Proportion	1 year; 2 years
	Sugary snack/drink taking frequency	Oral health behavior	Twice daily or more Once daily 2–6 times weekly Once or less weekly Never	Change from baseline	Proportion	1 year; 2 years
	Dental plaque	Oral hygiene status	Visible Plaque Index (VPI)	Change from baseline	Mean	1 year; 2 years

The primary outcome will be dental caries measured by dmft/dmfs (number of teeth/surfaces that are decayed, missing, or filled due to caries) of the children at age around 4 years (42–54 months). The children will be examined at the Prince Philip Dental Hospital (PPDH) or child care centers or kindergartens at baseline, 1 year, and 2 years follow-up.

The secondary outcomes will be as follows:

i. Average frequency of parental toothbrushing per day (2 times as preferred)
ii. Average frequency of intake of sugary snack/drink per day (2 times or less frequent as preferred)
iii. Oral hygiene status using the Visible Plaque Index (VPI), the presence or absence of plaque on the buccal and lingual surfaces of all primary teeth

#### Participant timeline {13}

The children will have an oral examination while the parents will self-complete a questionnaire at baseline before receiving their allocated intervention. The participants will be followed up after 1 and 2 years.

#### Sample size {14}

The sample size has been calculated using the G*Power software. From our recently completed clinical trial on family-centered oral health promotion for new parents and their infants, collected data show that the prevalence of ECC for children at 3 years old is 15% in the control group and 7% in the intervention group [24]. The reduction is 53%. In this study, we anticipate a smaller reduction, 40%, as the intervention will start when the young children are 18–30 months old instead of starting when the mothers were pregnant in our completed trial. Based on a previous study, the reported prevalence of dental caries of 4-year-old children in Hong Kong was 36% [3]. Assuming the prevalence of caries of children in our control group to be the same (i.e., 36%) and anticipating the prevalence of caries of children in the intervention group will be 22% (i.e., prevented fraction of 40%, Cohen’s *h* = 0.31, a small to moderate effect size, considered to be of clinical significance), the required sample size will be 164 child-parent dyads in each group for the 2-sided test at 0.05 level of significance and 80% power. Considering a typical value of ICC = 0.03 in a cluster randomized clinical trial setting and if 15–25 children are to be recruited from each cluster, the design effect (or variance inflation factor) will be 1.42–1.72 (= 1 + (25 − 1) × 0.03) [25]. Assuming a 10% dropout rate at the 2 years follow-up, the sample size will be increased to 259–314 in each group, thus 518–628 child-parent dyads altogether. Then, 26–36 clusters will be recruited. [This sample size is considered to be feasible as our research team has recently completed a randomized controlled trial that successfully recruited 692 parent-child dyads from 27 kindergartens with a 2-year follow-up rate of 91.3% [26].

#### Recruitment {15}

The staff in child care centers or kindergartens with nursery classes will help to recruit dyads with children aged 18–30 months. Researchers will try to balance the clusters by geographical and socio-demographic factors at recruitment. Both verbal persuasion and recruitment posters will be used for recruitment. Researchers will filter the dyads according to the eligibility criteria.

### Assignment of interventions: allocation

#### Sequence generation {16a}

The study adopts randomization at the cluster level. All participants in the same cluster will be assigned to the same study group (test group or control group). The randomized sequence for each study unit will be generated by Microsoft Excel.

#### Concealment mechanism {16b}

All randomization will be done before the intervention by a statistician not involved in the data collection. The allocation concealment will be ensured, as the statistician will not release the randomization code until the baseline examination has been completed.

#### Implementation {16c}

The number of study units will be allocated at a 1:1 ratio. After the allocation, the research assistant who is in charge of sending text messages will be told the assignments of the study units. The dental examiners will not be told about the allocation until the end of the study.

### Assignment of interventions: blinding

#### Who will be blinded {17a}

In the present study, the participants, the dental examiners, and the statisticians will be blinded. All parents will be blinded because they will receive the oral health mobile messages without telling them which group they will be assigned to. The dental examiners will be blinded to the group assignment of the study participants. The data submitted to the statisticians will only contain group A and group B for the group assignment so that the statisticians will be blinded when performing the data analysis.

#### Procedure for unblinding if needed {17b}

The study intervention is mobile messaging, which will not directly affect the participant’s health status, so there will be no unblinding procedure during the trial. Unblinding will be done at the end of the trial.

### Data collection and management

#### Plans for assessment and collection of outcomes {18a}

The data collection will involve dental examination and self-completed questionnaires. The children will have their dental examination at the PPDH or child care center/kindergarten (depending on the COVID-19 situation) at baseline, 1 year, and 2 years follow-up carried out by trained, experienced, and calibrated examiners (dentists who are postgraduate students in pediatric dentistry/dental public health under the supervision of an expert in pediatric dentistry in the research team). Methods, equipment, and indices as recommended by the World Health Organization (WHO) for conducting oral health surveys (WHO, 2013) will be employed. No X-ray will be taken. Dental behaviors during the examination will be observed and recorded.

The tooth status of the erupted primary teeth would be assessed by careful visual inspection. Dental caries assessment will be based on the merged ICDAS criteria (International Caries Detection and Assessment System), and lesions will be recorded as non-cavitated (codes 1 and 2) or cavitated (codes 3–6) caries [27]. Prior to the examination, the child’s teeth would be cleaned, and wet gauze would be used for the removal of food debris and dental plaque present on the tooth surfaces. No compressed air will be used in the examination because the children are too young to cooperate. The examiners would observe the occlusal, mesial, distal, buccal, and lingual surfaces of each tooth. Children in both the intervention group and the control group will be examined at the schools at baseline, 1 year, and 2 years follow-up. All the dental assessments will be charted by an assistant on a paper record form.

Self-completed questionnaires will be delivered to the parents at baseline, 1 year, and 2 years follow-ups by a link of a Google Form through mobile messaging. Information on the background and dental anxiety of the parents, HBM Scale, self-reported average tooth brushing frequency, sugar intake habits, and dental anxiety of their children will be collected. Possible confounding variables such as dental coverage scheme, dental visit history, dental treatment received, use of fluoride toothpaste, eating before bedtime, dental anxiety, HBM Scale, etc. will also be collected.

#### Plans to promote participant retention and complete follow-up {18b}

##### Retention

Once a dyad is enrolled, the study investigators will make every reasonable effort to follow the dyad for the entire study period.

The study investigators will maintain the interest of the parents in the study through text messages, incentivize parents by reminding them of a set of children’s oral health care products is to be received after the completion of the follow-up assessment, and give parents some feedback on their children’s oral health after each examination.

##### Data management {19}

The clinical charting form will be recorded in hard copy, and data will be entered into a computer by a research assistant. The EpiData software will be used for data entry. All the hard copies of the charting form will be kept in locked cabinets in Prince Philip Dental Hospital, which only can be accessed by the ethics committee (the Institutional Review Board of the University of Hong Kong/Hospital Authority Hong Kong West Cluster) and the research team. The research team members will check the charting forms every 3 months. The data on the charting form will be checked and corrected if there is anything wrong.

The questionnaire data will be collected in the form of Google Form, the parental responses will be downloaded every day and recorded in an Excel worksheet. If the parent forgot to fill out the questionnaire or filled out unreasonable information, he/she will be reminded via mobile messages to correct or amend the response at once. The information on the questionnaire will also be double-checked by the research team members regularly.

The data recorded in the charting forms and questionnaires will be permanently kept in the HKU Datahub e-platform. The ethics committees (e.g., HKU University Research Committee, Institutional Review Board of the University of Hong Kong/Hospital Authority Hong Kong West Cluster) and the research team will have access right to the data. The grouping information and parent contact numbers will be stored in electronic form with an authenticated password, which could only be accessed by the statistician who did the allocation and the research assistant in charge of the message delivery.

##### Confidentiality {27}

All hard copies of study-related information will be stored securely at the study site. All participant information will be stored in the computer with access only limited to the research team. The information of parents’ phone numbers and account numbers/names of WhatsApp and WeChat will only be used in the research.

#### Plans for collection, laboratory evaluation, and storage of biological specimens for genetic or molecular analysis in this trial/future use {33}

No biological specimens will be collected in this study.

## Statistical methods

### Statistical methods for primary and secondary outcomes {20a}

The effectiveness of HBM-based intervention will be evaluated by comparing the differences in the outcome variables between the intervention and control groups. Multilevel logistic regression adjusting for the effects of possible confounding factors for the clustered data will be performed to test the differences in the prevalence of dental caries [26] and the proportions of children with parental tooth brushing twice daily and sugar intake twice or less frequent per day between the two groups. Multilevel linear regression will be performed for the difference in the extent of dental caries (mean dmft/dmfs) and visible plaque level (mean %) between the two groups adjusted by other confounding factors for the clustered data [26]. The level of statistical significance for all tests will be set at 0.05. Two-level random-intercept models will be considered: children as level 1 and schools as level 2. All the analyses will be performed using the SPSS software package.

### Interim analyses {21b}

The present intervention of the study is the oral health educational message, which would not arouse any harm to the participants. Besides, the data recorded in the charting forms and questionnaires will be checked and proofread regularly. Therefore, interim analyses will not be needed in this study.

### Methods for additional analyses (e.g., subgroup analyses) {20b}

There are no plans for additional analyses.

### Methods in analysis to handle protocol non-adherence and any statistical methods to handle missing data {20c}

The intention-to-treat approach will be used for data analysis based on the random allocation regardless of whether the intervention has been received or not. Reasons for any non-adherence or withdrawal will be recorded for the subsequent analysis. Missing data will be checked, and where appropriate, multiple imputations will be used and sensitivity analyses will be conducted.

### Plans to give access to the full protocol, participant-level data, and statistical code {31c}

The protocol has been uploaded on ClinicalTrials.gov (ID: NCT04665219). The participant-level data will be uploaded to the DataHub e-platform of The University of Hong Kong.

### Oversight and monitoring

#### Composition of the coordinating center and trial steering committee {6a}

The principal investigator is responsible for the design of the study and the coordination of different cooperation partners. The research team comprises the trial steering committee responsible for the recruitment, dental check-up, message delivery, and data analysis.

#### Composition of the data monitoring committee, its role, and reporting structure {21a}

Adverse effects of oral health promotion as an intervention are not anticipated; thus, no data monitoring committee is needed in this study.

#### Adverse event reporting and harms {22}

Adverse effects of oral health promotion as an intervention are not anticipated. However, suppose any adverse events are to have occurred, the parents would be reminded to report to the research team via mobile message. The adverse events will be reported to the Institutional Review Board of the University of Hong Kong/Hospital Authority Hong Kong West Cluster.

#### Frequency and plans for auditing trial conduct {23}

The trial steering committee will report the progress of the study to the Institutional Review Board of the University of Hong Kong/Hospital Authority Hong Kong West Cluster annually and will report the findings at the end of the study.

#### Plans for communicating important protocol amendments to relevant parties (e.g., trial participants, ethical committees) {25}

If there would be any further necessary protocol amendments, approvals will be sought from the research grant committee of the Health and Medical Research Fund, Food and Health Bureau (FHB), Government of Hong Kong SAR, and the ethical committee (Institutional Review Board of the University of Hong Kong/Hospital Authority Hong Kong West Cluster). The trial participants will be notified as well.

#### Dissemination plans {31a}

The research findings will be published in international peer-reviewed journals. Besides the publications, we plan to communicate the research findings with the Chief Dental Officer and his team at the Department of Health in the government. We also plan to share the findings with local dental professional bodies (e.g., Hong Kong Dental Association, Hong Kong Society of Paediatric Dentistry, Hong Kong Paediatric Society, etc.), dental and medical practitioners, and dental hygienists and medical nurses working in child health care setting.

## Discussion

With the rapid spread of mobile technology around the globe, mobile health (mHealth) has become increasingly popular in the past few years. According to the third global survey of WHO Global Observatory for eHealth, the use of mHealth has kept growing since 2010 [28]. By offering care at a distance and mutual communication, mHealth service could reach remote populations, even those in underserved communities, enabling greater equity in universal health coverage [29, 30].

There have been some mHealth practices in the field of oral health care field [31–33]. Text messages, health care helplines, webpages, and mobile apps have been frequently used in these mHealth programs, among which the mobile information and communication technology was most adopted [28]. The information can be conveyed via text, pictures, or multimedia. Although most investigators claimed that their program could successfully promote a good oral health attitude and behavior among the targeted population, few were empirically validated to demonstrate their effectiveness. Tiffany et al. identified 19 available mobile apps for oral health promotion designed for Android or iOS in 2018, with findings that the content of oral health care was unprofessional, and the design of most apps was not driven by a sound behavioral theory [34].

In the present study, HBM is selected as the framework for text messages. HBM is recommended as a useful theoretical model to explain health behaviors, and it is also useful to plan behavioral interventions [18]. HBM emphasizes individual characteristics and cognitive factors, giving less attention to social influences and emotional components of behavior [35]. HBM suggests that health-promoting behaviors can be triggered by the presence of six domains: perceived susceptibility, perceived severity, perceived barriers, perceived benefit, cue to action, and perceived self-efficacy [18]. It is reported that intervention of psychological theories based on HBM could effectively improve oral health behaviors [19]. On the other hand, evidence also showed that HBM-based intervention effectively improves adherence to oral health care instructions among adults and school children [36].

Previously, the emphasis of HBM-based health behavior intervention would be laid on susceptibility, severity, and benefits. The domains of “cue to action” and “self-efficacy” were less addressed [37], and barriers were not usually proactively expressed by subjects. In the present study, a set of standardized messages will be sent to address HBM domains of susceptibility, severity, and benefit. Besides, parents will be encouraged to share their concerns/experiences/thoughts via texting us back, which will be responded/discussed/solved promptly. With these interactive messages, the barriers could be addressed in a timely manner. They could receive enough cues to action, so that continuous support can be provided to facilitate the enhancement of parental self-efficacy, through which positive actions are likely to occur.

Dental caries is a multifactor disease [38], whose risk factors are diverse and, like most non-communicable diseases, related to social-economic factors [1]. Therefore, parents’ oral health behavior is not the only associated factor of ECC. To eliminate the influence of the confounding factors, we will collect information on possible confounding variables and include them in the multivariate analysis.

Caries prevention projects among children involving oral health education are often delivered through primary care, home visits, or kindergarten-based programs [39–41]. However, most of these strategies have resulted in small, clinically insignificant effects on caries prevention. By HBM-based intervention via a low-cost text messaging vehicle, the caries prevention scheme may help the parents establish proper oral health behaviors for their children and

## Trial status

The protocol is version no. 3, dated 2021.03.30. The recruitment began on 2020.10.06, and it is still ongoing.

## Supplementary Information

**Additional file 1.** Participants informed consent form.

## Data Availability

A completely de-identified data set will be uploaded to the DataHub of The University of Hong Kong for sharing.

## Abbreviations

HBM: Health belief model
SMS: Sending text messages
ECC: Early childhood caries
RCT: Randomized controlled trial
mHealth: Mobile health
ICC: Intracluster correlation coefficient
CPI: Community Periodontal Index
ICDAS: International Caries Detection and Assessment System
VPI: Visible Plaque Index
