# Supplementary material for: Health belief model for empowering parental toothbrushing and sugar intake control in reducing early childhood caries among young children—study protocol for a cluster randomized controlled trial
Source: Trials. 2022 Apr 12;23:298. doi: 10.1186/s13063-022-06208-w (PMC9003160; doi:10.1186/s13063-022-06208-w)
Supplement: Supplementary file 1 — Additional file 1. Participants informed consent form. [file 13063_2022_6208_MOESM1_ESM.docx]

| Faculty of Dentistry  The Prince Philip Dental Hospital  34 Hospital Road  Hong Kong  Tel No.: (852) 2859 0295  Fax No: (852) 2858 9464 | 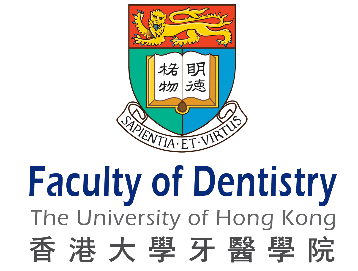 | **香港大學牙醫學院**  香港西營盤醫院道34號  菲臘牙科醫院 |
| --- | --- | --- |

**PARTICIPANTS INFORMED CONSENT FORM**

**參與者知情同意書**

Participant Identification Number 參與者識別號碼: _____________________

**Title of Project: Is Health Belief Model effective in preventing tooth decay in young children?**

**研究項目： 健康信念模式能有效預防幼兒蛀牙？**

**Researcher研究者: Prof May Wong 王春美教授，Dr Gillian Lee 李曉雯醫生, Dr Xiaoli Gao 高曉莉醫生，**

**Dr Pei Liu 劉沛醫生, Prof Samuel Wong 黃仰山教授**

|  | | **Please tick box**  **請勾選方格** |
| --- | --- | --- |
| 1. | I confirm that I have read and understood the information sheet dated 24 March 2021 (version 3.0) for the above study and have the opportunity to ask questions.  本人確認已閱讀並理解有關上述研究於2021年3⽉24⽇準備的資料單張(版本3.0)，並有機會提問。 | □ |
| 2. | I understand that my child and my participation are voluntary and that my child and I are free to withdraw at any time, without giving any reason, without the care or legal rights of my child being affected.  我了解參與上述研究完全是自願的。我/我的⼦女能在任何時候退出，不必提供任何理由。退出上述研究，不會影響到我/我的子女的牙科治療或法律權益。 | □ |
| 3. | I understand that sections of any of the medical notes of my child may be looked at by responsible individuals from the University of Hong Kong, Institutional Review Board of the University of Hong Kong / Hospital Authority Hong Kong West Cluster or from regulatory authorities where it is relevant to his/her taking part in research or for ethics review purpose. I give permission for these individuals to have access to my child records.  我了解我的子女與上述研究有關的任何醫療記錄會予香港⼤學、香港大學及醫管局港島西醫院聯網研究倫理委員會或管制當局的負責人查閱。我允許這些⼈士使⽤我子女的醫療記錄。 | □ |
| 4. | I agree to take part in the above study.  我同意參與上述的研究。 | □ |
| 5. | I agree to let my child, ___________________________, to take part in the above study.  我同意讓我的子女 ── ___________________________，參與上述的研究。 | □ |

| Name of Parent/Guardian | Date | Signature |
| --- | --- | --- |
| 家長/監護人姓名 | 日期 | 簽名 |

| Name of Witness (if applicable) | Date | Signature |
| --- | --- | --- |
| 見證人姓名（如適用） | 日期 | 簽名 |

| Name of Person taking consent /Researcher | Date  日期 | Signature  簽名 |
| --- | --- | --- |
| 獲得同意者/研究員姓名 |  |  |

Copies to副本:• Participant參與者 & Researchers’ file研究者檔案
